# Thrombus aspiration during primary percutaneous coronary intervention is associated with reduced myocardial edema, hemorrhage, microvascular obstruction and left ventricular remodeling

**DOI:** 10.1186/1532-429X-14-19

**Published:** 2012-03-26

**Authors:** Mohammad I Zia, Nilesh R Ghugre, Kim A Connelly, Subodh B Joshi, Bradley H Strauss, Eric A Cohen, Graham A Wright, Alexander J Dick

**Affiliations:** 1 Division of Cardiology, Schulich Heart Centre, Sunnybrook Health Sciences Centre, University of Toronto, 2075 Bayview Avenue, Toronto, ON M4N 3 M5, Canada

**Keywords:** Thrombus aspiration, Cardiovascular magnetic resonance, Myocardial infarction

## Abstract

**Background:**

Thrombus aspiration (TA) has been shown to improve microvascular perfusion during primary percutaneous coronary intervention (PCI) for patients with ST-segment elevation myocardial infarction (STEMI). The objective of our study was to assess the relationship between TA and myocardial edema, myocardial hemorrhage, microvascular obstruction (MVO) and left ventricular remodeling in STEMI patients using cardiovascular magnetic resonance (CMR).

**Methods:**

Sixty patients were enrolled post primary PCI and underwent CMR on a 1.5 T scanner at 48 hours and 6 months. Patients were retrospectively stratified into 2 groups: those that received TA (35 patients) versus that did not receive thrombus aspiration (NTA) (25 patients). Myocardial edema and myocardial hemorrhage were assessed by T2 and T2* quantification respectively. MVO was assessed via a contrast-enhanced T1-weighted inversion recovery gradient-echo sequence.

**Results:**

At 48 hours, infarct segment T2 (NTA 57.9 ms vs. TA 52.1 ms, p = 0.022) was lower in the TA group. Also, infarct segment T2* was higher in the TA group (NTA 29.3 ms vs. TA 37.8 ms, p = 0.007). MVO incidence was lower in the TA group (NTA 88% vs. TA 54%, p = 0.013).

At 6 months, left ventricular end-diastolic volume index (NTA 91.9 ml/m2 vs. TA 68.3 ml/m2, p = 0.013) and left ventricular end systolic volume index (NTA 52.1 ml/m2 vs. TA 32.4 ml/m2, p = 0.008) were lower and infarct segment systolic wall thickening was higher in the TA group (NTA 3.5% vs. TA 74.8%, p = 0.003).

**Conclusion:**

TA during primary PCI is associated with reduced myocardial edema, myocardial hemorrhage, left ventricular remodeling and incidence of MVO after STEMI.

## Background

Primary percutaneous coronary intervention (PCI) is the mainstay of treatment in patients with ST-segment elevation myocardial infarction (STEMI) [1]. Despite advances in primary PCI and medical therapy, the incidence of heart failure, re-infarction and death in these patients remains significant [2]. No-reflow is a common adverse event leading to worse clinical outcomes in patients with STEMI undergoing primary PCI, with atherothrombotic embolization being one of the contributing mechanisms [3-6]. Thrombus aspiration (TA) can theoretically protect the microcirculation from distal embolization, however randomized clinical trials have resulted in conflicting results [4,7-21]. In the study by Kaltoft et al. [13], thrombectomy was associated with increased infarct size and did not improve ST-segment resolution. Conversely, other trials such as REMEDIA (Randomized Evaluation of the Effect of Mechanical Reduction of Distal Embolization by Thrombus-Aspiration in Primary and Rescue Angioplasty), DEAR-MI (Dethrombosis to Enhance Acute Reperfusion in Myocardial Infarction) and TAPAS (Thrombus Aspiration During Percutaneous Coronary Intervention in Acute Myocardial Infarction) showed that thrombectomy improved microvascular perfusion. However, the impact of TA on other parameters of microvascular injury such as myocardial edema and hemorrhage was not addressed in any of these trials.

Cardiovascular magnetic resonance (CMR) represents the gold-standard method in assessing myocardial edema, myocardial hemorrhage, left ventricular remodeling and microvascular obstruction (MVO) [22-24]. The purpose of this study was to evaluate the relationship between TA as adjunctive therapy in primary PCI for patients with STEMI, and myocardial edema, myocardial hemorrhage, left ventricular remodeling and MVO.

## Methods

### Study population

Sixty patients presenting to Sunnybrook Health Sciences Centre in Toronto, with STEMI between July 2009 and March 2011 were enrolled. The main inclusion criteria were patients that met the standard diagnostic criteria for STEMI [25]. All patients had undergone primary PCI as part of a regional program that triages patients with STEMI to our centre for early revascularization. Exclusion criteria included hemodynamic instability (defined as systolic blood pressure < 90 mmHg, use of inotropic agents or an intraortic balloon pump), significant arrhythmias, significant renal dysfunction (estimated glomerular filtration rate < 30 mL/min), and typical contraindications to CMR such as pacemakers and implantable cardioverter-defibrillators. We obtained written consent from all patients, and the study was approved by the ethics review board of Sunnybrook Health Sciences Centre.

### Primary PCI protocol

All patients were pretreated prior to revascularization with aspirin 162 mg and clopidogrel 600 mg. Choice of anticoagulant (intravenous heparin or bivalirudin) and optional use of glycoprotein IIb/IIIa inhibitor were left to operator discretion. TA was performed with an Export Medtronic device (Medtronic Inc., Minneapolis, Minnesota) and its use was also left to operator discretion. Factors influencing the use of TA included target vessel size, degree of thrombus burden, no reflow phenomenon and clinical instability. Subsequently patients received aspirin, clopidogrel, beta-blockers, angiotensin-converting enzyme inhibitors, angiotensin-receptor blockers and statins as per standard of care.

### Angiographic and electrocardiographic analysis

Thrombolysis In Myocardial Infarction (TIMI) flow grade, myocardial blush grade and thrombus score were estimated visually using previously described methods by 2 experienced observers who were blinded to use of TA [26-28]. ST-segment resolution, defined as ≥70% reduction in the sum of the ST-segment elevation score between electrocardiograms obtained before primary PCI and immediately after the procedure, was also recorded [29].

### CMR acquisition protocol

Each patient was imaged at 2 time points: within 48 hours and 6 months post STEMI. Studies were performed on a 1.5 T clinical scanner (Signa Twinspeed HDx, GE Healthcare, Waukesha, WI) with commercially available CMR software, electrocardiographic triggering, 8-channel receive coil and breath holds at end-expiration. After performing a localizer scan, short-axis cine CMR (10-12 slices) was performed with a balanced steady-state-free-precession sequence (FIESTA, GE Healthcare) with the following parameters: repetition time = 3.7 ms and echo time = 1.6 ms, flip angle = 45°, slice thickness = 8 mm, acquisition matrix = 256 × 192, field of view = 35 cm, bandwidth = 125 kHz, 20 cardiac phases per slice.

Myocardial edema was assessed by T2 quantification using a previously validated free-breathing cardiac-gated spiral imaging sequence with T2 preparation [30,31], 8-10 contiguous slices from base to apex and the following typical parameters: echo times = 2.9, 24.3, 88.2, 184.2 ms, 12 spiral interleaves with 4096 points each (16.4 ms duration), slice thickness = 6 mm, bandwidth = 125 kHz. Myocardial hemorrhage was assessed by T2* quantification using a cardiac-gated spoiled gradient echo sequence with multiple echoes, 8-10 contiguous slices from base to apex, and the following typical parameters: 8 echoes (1.4-12.7 ms), repetition time = 14.6 ms, flip angle = 30°, acquisition matrix = 128 × 128, 8 views-per-segment, slice thickness = 8 mm, bandwidth = 100 kHz [31]. All echoes for a single slice were acquired in a single breath hold. Finally, a breath-hold, contrast-enhanced T1-weighted inversion recovery gradient-echo sequence was used to depict the presence, location, and extent of myocardial infarction and MVO using the following parameters: repetition time/echo time = 5.4/2.5 ms, flip angle = 30°, 8-10 contiguous slices from base to apex, slice thickness = 8 mm, acquisition matrix = 192 × 128, field of view = 35 cm. The inversion time was manually adjusted to null signal from normal myocardium. Short axis images were obtained 10 minutes after intravenous administration of Gadolinium-diethylene triamine pentaacetic acid (0.2 mmol/kg; Gadovist, Bayer Healthcare, Wayne, NJ).

### CMR image analysis

All cine CMR images were analyzed using QMass^® ^MR software (Medis, Netherlands), and the results were the consensus of a minimum of 2 experienced observers who were blinded to use of TA. Endocardial and epicardial borders were delineated on the end-diastolic and end-systolic phases of short-axis slices for quantification of left ventricular volumes, function, mass, and infarct segment systolic wall thickening. Measurements were indexed for body surface area. End-diastolic wall thickness was measured in the infarct segment as an indicator of myocardial edema in the acute phase and wall thinning in the chronic phase. Myocardial T2 and T2* maps were constructed by fitting signal intensities at each pixel with an exponential model for the given echo times. Hemorrhage was identified as an area of hypointensity on T2-weighted images within the infarcted segment. Infarct size was quantified in a contrast-enhanced T1-weighted image using the full-width-half-maximum technique as previously described [32] while MVO was identified as a region of hypoenhancement within the region of the hyperenhanced infarct, 10 minutes post contrast administration. Infarct size, hemorrhage, and MVO were expressed as a percentage of total myocardium. Data fitting for T2/T2* maps, infarct, hemorrhage and MVO size computation were performed with custom-written scripts developed in MATLAB^® ^(The Mathworks, Natick, MA). MVO was also recorded as a binary variable of being present or absent, based on the contrast-enhanced images [33].

### Statistical analysis and endpoints

The study population was divided into 2 groups: TA versus no thrombus aspiration (NTA). Primary study end points were the degree of myocardial edema (T2 and end-diastolic wall thickness measurements), myocardial hemorrhage (T2* measurement) and occurrence of MVO at the first time point of within 48 hours post-STEMI. Secondary endpoints included the occurrence of final myocardial blush grade ≥ 2, final TIMI flow ≥ 2 on angiography, rate of ST-segment resolution post primary PCI, and parameters of left ventricular remodeling including left ventricular end diastolic volume index (LVEDI), left ventricular end systolic volume index (LVESI), left ventricular stroke volume index (LVSVI), left ventricular ejection fraction, and infarct segment systolic wall thickening at the final time point of 6 months post STEMI. Continuous data were expressed as mean +/- standard deviation and analyzed by the Student *t *test. Categorical variables were reported as frequencies and percentages and analyzed by the chi-square or Fisher exact test, as appropriate. All tests were 2-tailed, and statistical significance was accepted at p < 0.05.

## Results

### Baseline characteristics

Eighty-five patients with STEMI were screened, 25 patients were excluded and 60 patients were included in the study. Reasons for exclusion included severe renal insufficiency, claustrophobia, hemodynamic instability and presence of permanent pacemaker. Clinical characteristics of both the TA and NTA group are shown in Table 1 and appear to be well matched in both groups. No difference in symptom to balloon time or door to balloon time was observed between groups. Reasons for not using TA by the operators included minimal thrombus burden visualized on the coronary angiogram, small vessel size, or preserved distal flow throughout the procedure. Glycoprotein IIb/IIIa inhibitor use was similar in both groups (NTA 52% vs. TA 62.9%, p = 0.514).

**Table 1:** Baseline Clinical Characteristics of the Study Population

	Total (n = 60)	NTA (n = 25)	TA (n = 35)	p Value
Age, yrs	59.6 ± 10.6	60.0 ± 7.3	59.5 ± 12.6	0.741

Males (%)	53 (88.3)	21 (84.0)	32 (91.4)	0.273

Diabetes (%)	15 (25.0)	6 (24.0)	9 (25.7)	0.242

Hypertension (%)	27 (45.0)	12 (48.0)	15 (42.9)	0.312

Dyslipidemia (%)	15 (25.0)	7 (28.0)	8 (22.9)	0.414

Smoking (%)	17 (28.3)	8 (32.0)	9 (25.7)	0.372

Prior Myocardial Infarction (%)	2 (3.3)	1 (4.0)	1 (2.9)	0.718

Prior PCI (%)	4 (6.7)	1 (4.0)	3 (8.6)	0.384

Creatinine, umol/L	82.1 ± 23.0	81.1 ± 17.1	82.6 ± 24.3	0.743

Symptoms to balloon, minutes	397 ± 304	402 ± 268	391 ± 291	0.831

Door to balloon, minutes	83.5 ± 51.2	83.2 ± 39.8	83.9 ± 59.8	0.765

Abbreviations. *NTA *no thrombus aspiration; *PCI *percutaneous coronary intervention; *TA *thrombus aspiration

### Angiographic, periprocedural and electrocardiographic findings

TA was associated with more frequent final myocardial blush grade ≥ 2 (NTA 44% vs. TA 88.6%, p < 0.01) and ST-segment resolution (NTA 40% vs. TA 68.6%, p = 0.036) post primary PCI (Table 2). Final TIMI flow ≥ 2 was equivalent in both groups (NTA 96% vs. TA 97.1%, p = NS).

**Table 2:** Peri-Procedural Characteristics and Outcomes of the Study Population

	Total (n = 60)	NTA (n = 25)	TA (n = 35)	p Value
Infarct artery (%)				
LAD	26 (43.3)	12 (48.0)	14 (40.0)	0.344
LCx	12 (20.0)	6 (24.0)	6 (17.1)	0.210
RCA	22 (36.7)	9 (36.0)	13 (37.1)	0.416

Multivessel disease (%)	26 (43.3)	9 (36.0)	17 (48.6)	0.128

Thrombus score				
2	22 (36.7)	8 (32.0)	14 (40.0)	0.224
3	23 (38.3)	11 (44.0)	12 (34.3)	0.419
4	4 (6.7)	2 (8.0)	2 (5.7)	0.426

Adjunctive Pharmacologic Therapy				
Glycoprotein IIb/IIIa inhibitor (%)	35 (58.3)	13 (52.0)	22 (62.9)	0.514
Bivalirudin (%)	25 (41.7)	11 (44.0)	14 (40.0)	0.664

Stent type				
Bare-metal	41 (68.3)	16 (64.0)	25 (71.4)	0.517
Drug-eluting	19 (31.7)	8 (32.0)	11 (31.4)	0.736

Mean peak creatine kinase peak (IU/L)	2177 ± 1579	2321 ± 1271	2142 ± 1643	0.513

ST-segment resolution (%)	34 (56.7)	10 (40.0)	24 (68.6)	0.036

Post PCI TIMI flow grade (%)				
> 2	58 (96.7)	24 (96.0)	34 (97.1)	0.987

Post PCI MBG (%)				
> 2	42 (70.0)	11 (44.0)	31 88.6)	0.001

Mean PCI to CMR time				
Scan 1, hours	34.4 ± 17.3	36.6 ± 16.2	34.0 ± 17.6	0.435
Scan 2, days	243 ± 62	222 ± 52	247 ± 69	0.212

Abbreviations. *CMR *cardiovascular magnetic resonance; *LAD *left anterior descending; *LCx *left circumflex; *MBG *myocardial blush grade; *RCA *right coronary artery; *TIMI *Thrombolysis in Myocardial Infarction; other abbreviations as in Table 1

### CMR analysis

The initial CMR scan was performed at a mean of 34.4 hours post primary PCI and the second scan at a mean of 243 days post primary PCI in the overall group. Table 3 shows the CMR outcomes for both time points.

**Table 3:** Cardiac Magnetic Resonance Imaging Outcomes in Study Population

	Scan 1 (48 Hours)			Scan 2 (6 Months)		
	**NTA**	**TA**	**p Value**	**NTA**	**TA**	**p Value**

IS T2, ms	57.9 ± 7.9	52.1 ± 6.3	0.022	39.5 ± 3.0	40.6 ± 2.8	0.517

RS T2, ms	41.9 ± 2.7	42.4 ± 2.8	0.552	39.1 ± 2.9	39.6 ± 2.7	0.612

IS EDWT, mm	11.4 ± 2.2	9.3 ± 1.6	0.013	4.0 ± 1.1	5.7 ± 1.0	0.008

RS EDWT, mm	7.0 ± 1.4	6.9 ± 1.3	0.883	8.3 ± 1.3	7.8 ± 1.2	0.443

IS T2*, ms	29.3 ± 9.4	37.8 ± 7.0	0.007	33.9 ± 4.9	35.7 ± 5.7	0.435

RS T2*, ms	37.1 ± 3.4	38.0 ± 3.1	0.662	38.0 ± 3.7	38.2 ± 4.4	0.712

Hemorrhage, % of myocardium	11.6 ± 6.8	7.1 ± 3.8	0.02	-	-	-

Hemorrhage, n (%)	13 (52.0)	7 (20.0)	0.02	-	-	-

MVO, % of myocardium	6.7 ± 4.2	3.4 +3.1	0.003	-	-	-

MVO, n (%)	22 (88.0)	19 (54.3)	0.013	-	-	-

Infarct Size, % of myocardium	27.3 ± 9.1	19.8 ± 8.2	0.001	16.8 ± 8.7	13.8 ± 4.8	0.022

LVEDVI, ml/mm2	68.6 ± 17.6	70.4 ± 11.9	0.717	91.9 ± 21.1	68.3 ± 8.7	0.013

LVESVI, ml/mm2	37.1 ± 12.3	38.1 ± 9.9	0.873	52.1 ± 16.9	32.4 ± 5.3	0.008

LVSVI, ml/mm2	30.4 ± 9.5	32.7 ± 4.3	0.546	40.4 ± 11.2	35.8 ± 8.1	0.301

LVEF, %	44.7 ± 8.1	46.4 ± 5.9	0.392	46.8 ± 10.5	53.8 ± 7.7	0.11

IS-SWT, %	4.9 ± 24.8	-2.9 ± 18.3	0.224	3.5 ± 19.5	74.8 ± 58.5	0.003

Abbreviations. *EDWT *end diastolic wall thickness; *IS *infarct segment; *IS-SWT *infarct segment systolic wall thickening; *LVEDVI *left ventricular end-diastolic volume index; *LVEF *left ventricular ejection fraction; *LVESVI *left ventricular end-systolic volume index; *LVSVI *left ventricular stroke volume index; *MVO *microvascular obstruction; *RS *remote segment; other abbreviations as in Tables 1 and 2

**Figure 1:**
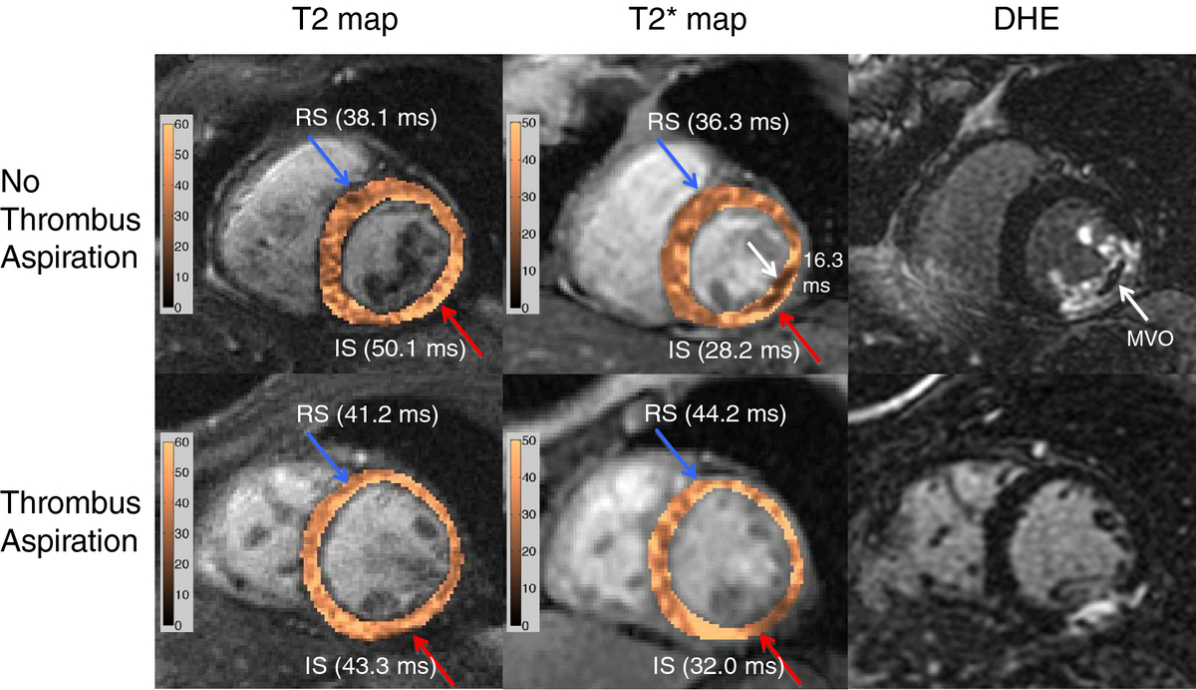
Cardiac Magnetic Resonance Imaging Scan of Patients From Both Study Groups, No Thrombus Aspiration (NTA) and Thrombus Aspiration (TA) at 48 Hours: show myocardial T2 and T2* maps along with late gadolinium enhancement (LGE) in short-axis views from representative patients in the NTA and TA groups. T2 and T2* maps of the myocardium have been overlaid on T2-weighted and T2*-weighted images. The red and blue arrows represent infarct segment (IS) and remote segment (RS) in the myocardium, respectively. Note that edema was greater (higher T2) in the NTA patient compared to the TA patient in the IS. Lower T2* within the infarct core (white arrow) in the NTA patient was indicative of myocardial hemorrhage and was associated with microvascular obstruction (MVO) observed on the LGE image (white arrow)

Figure 1 demonstrates the myocardial T2 and T2* maps along with late gadolinium enhancement (LGE) images in short-axis views from representative patients in the NTA and TA groups at 48 hours. In this acute phase, both measures of myocardial edema, i.e. infarct segment T2 (NTA 57.9 ms vs. TA 52.1 ms, p = 0.022) and infarct segment end-diastolic wall thickness (NTA 11.4 mm vs. TA 9.3 mm, p < 0.01), were lower in the TA group (Table 3). Infarct segment T2* was higher, indicative of reduced myocardial hemorrhage, in the TA group (NTA 29.3 ms vs. TA 37.8 ms, p = 0.007). MVO incidence was also lower in the TA group (NTA 88% vs. TA 54.3%, p = 0.013).

**Figure 2:**
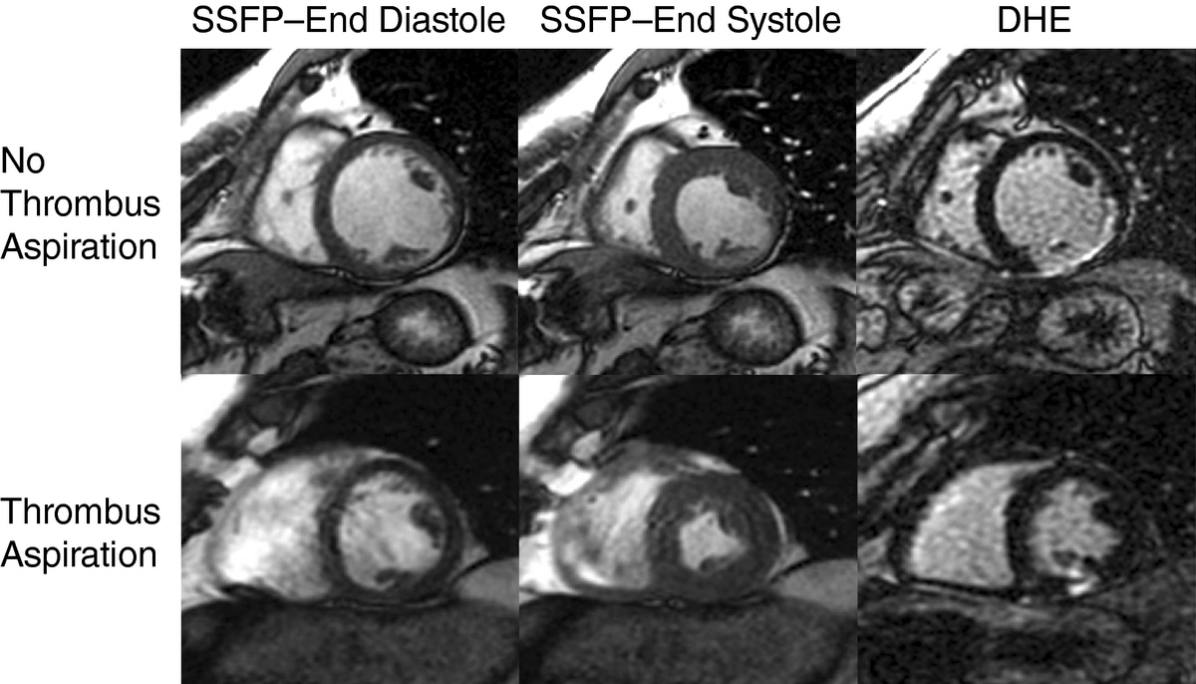
**Cardiac Magnetic Resonance Imaging Scan of Patients From Both Study Groups, No Thrombus Aspiration (NTA) and Thrombus Aspiration (TA) at 6 Months: Images show a short axis slice with left ventricular end diastolic volume index (LVEDVI) measurement in first column**. Short axis slices in the second column highlight the difference in left ventricular end systolic volume index (LVESVI) in the 2 treatment groups. Late gadolinium enhancement (LGE) images in the last column demonstrate the location of the infarct.

Figure 2 demonstrates the LVEDVI and LVESVI measurements along with LGE images in short-axis views from patients in both treatment groups at 6 months. Baseline LVEDVI, LVESVI, LVSVI, infarct segment systolic wall thickening, and left ventricular ejection fraction measurements were similar in both treatment groups. At 6 months, LVEDVI (NTA 91.9 ml/m2 vs. TA 68.3 ml/m2, p = 0.013) and LVESVI (NTA 52.1 ml/m2 vs. TA 32.4 ml/m2, p = 0.008) were lower in the TA group (Table 3). LVSVI (NTA 40.4 ml/m2 vs. TA 35.8 ml/m2, p = 0.30) and left ventricular ejection fraction (NTA 46.8% vs. TA 53.8%, p = 0.11) were equivalent in both groups. Infarct segment systolic wall thickening (NTA 3.5% vs. TA 74.8%, p = 0.003) and end diastolic wall thickness (NTA 4 mm vs. TA 5.7 mm, p = 0.008) were both greater in the TA group at 6 months.

## Discussion

Our non-randomized study demonstrates that TA during primary PCI is associated with reduced myocardial edema, myocardial hemorrhage, infarct size, left ventricular remodeling, and MVO after STEMI. We postulate that TA results in less myocardial injury, evidenced by the reduced infarct size, which in turn is responsible for reduced myocardial edema and hemorrhage in the acute phase. This is the first study to use quantitative CMR parameters to evaluate the effects of TA on myocardial edema and myocardial hemorrhage. Recently, the utility of quantitative CMR in the longitudinal evaluation of myocardial edema, hemorrhage, and MVO has been demonstrated in preclinical models of acute myocardial infarction [31].

Both myocardial hemorrhage and MVO have been extensively linked to worse clinical outcome post acute myocardial infarction [22,24,33,34]. Recently, the EXPIRA trial [17] had shown that thrombectomy reduced the extent of MVO and infarct size after acute myocardial infarction, which is consistent with our findings. Our study had an even higher incidence of MVO in the NTA group compared to the EXPIRA trial (88% vs. 72.9%); however, the absolute difference between the 2 treatment groups was similar in both studies (34% vs. 41.4%).

Myocardial hemorrhage is considered to be a sign of severe microvascular injury [35]. Histologically, it is characterized by vascular cell damage, with leakage of red blood cells from injured cells affecting mainly the mid-myocardial layer [36]. It is a frequent complication after successful myocardial reperfusion, affecting 25% of patients, and is an independent predictor of adverse left ventricular remodeling regardless of the initial infarct size, and a marker of late arrhythmic risk [22,31,34]. The pathophysiology relating MVO and myocardial hemorrhage is unclear. It is possible that MVO leads to endothelial damage and subsequent extravasation of erythrocytes to the interstitium. However, it has also been postulated that the myocardial hemorrhage causes swelling of the myocardium and subsequent compression of the microvasculature, thereby actually causing or worsening MVO [37,38]. While the mechanism is uncertain, the fact that TA is associated with a lower incidence of MVO and reduced T2* indicating reduced myocardial hemorrhage points to the utility of this adjunctive therapy in primary PCI. Mechanical thrombus removal may explain the better results obtained in the TA group.

Extensive preclinical and human studies have established that T2 signal hyperintensity by CMR indicates increased myocardial water content [39,40]. T2 may increase within 30 minutes of ischemia onset, even before detectable injury by troponin or late gadolinium enhancement [41,42]. Also, end-diastolic wall thickness has been shown to be able to identify and monitor the presence, extent and resolution of myocardial edema following primary PCI [43,44]. The presence of myocardial edema after an acute coronary syndrome has been recently shown to be associated with a higher hazard of cardiovascular event or death within 6 months [45]. Hence, our observation that TA during primary PCI is associated with reduced myocardial edema has important clinical implications and can explain why 2 previous randomized clinical trials have shown a reduction in major adverse cardiac events in the thrombectomy group [17,21].

We used a quantitative T2 mapping technique to delineate the extent of myocardial edema instead of a T2-weighted imaging approach. T2-weighted imaging techniques have known limitations, which impairs their validity [39,46]. Some of these limitations include: a) surface coil intensity inhomogeneity leading to variability in myocardial signal, b) sub-endocardial bright signal artifact caused by stagnant blood, c) cardiac motion leading to reduced myocardial signal, d) subjective nature of qualitative T2-weighted imaging assessment which then poses significant challenges in tracking longitudinal changes in a robust manner. Recently other studies have demonstrated that quantitative T2 mapping addresses the known problems associated with T2-weighted imaging and can offer increased accuracy in the detection of myocardial edema [31,47-50].

Hemorrhage is known to not only reduce T2* values but also T2 relaxation times [50]. An unexpected finding in our study is that patents in the NTA group had increased T2 relaxation times despite having more hemorrhage. We postulate that patients in the NTA group had more hemorrhage, more extensive myocardial injury and therefore more edema. The fact that end-diastolic wall thickness was increased acutely in the infarct segment of the NTA group supports our hypothesis that these patients indeed have more edema. In fact, we may have obtained an even higher T2 value in NTA patients if susceptibility effects of hemorrhage were not present.

In contrast to the EXPIRA [17] trial that had CMR follow up at 3 months, we assessed left ventricular remodeling at 6 months when infarct expansion is well established. Using left ventricular remodeling parameters indexed to body surface area we were able to demonstrate a significant improvement in LVEDVI, LVESVI and infarct segment systolic wall thickening in the TA group. Two prior studies using echocardiography have had conflicting results with respect to left ventricular remodeling and use of TA. De Luca and colleagues showed that in 76 patients with anterior myocardial infarction, TA was associated with significantly lower end-diastolic and end-systolic left ventricular volumes on transthoracic echocardiography at 6 months compared to conventional primary PCI [11]. In contrast, Galiuto et al. showed no significant difference in left ventricular volumes at 6 months with thrombectomy using myocardial contrast echocardiography [51]. Our significant findings in all types of infarcts, can perhaps be explained by the superiority of CMR over conventional echocardiography and myocardial contrast echocardiography in terms of spatial resolution and endocardial definition [52]. In addition, we were able to demonstrate that TA is associated with reduced myocardial hemorrhage, which in turn is an independent predictor of adverse left ventricular remodeling [34].

In accordance with the literature, TA also improved final myocardial blush grade and the rate of ST-segment resolution post primary PCI [11,17,18,20]. The degree of improvement in both these parameters of microvascular integrity was comparable to previous studies.

### Study limitations

This study represents a single-centre non-randomized experience with a limited number of patients. However, baseline demographic and angiographic characteristics appear to be well matched in our 2 study groups. In particular, time to reperfusion, culprit vessel distribution, thrombus burden, enzymatic infarct size and adjunctive glycoprotein IIb/IIIa inhibitor use were similar in both groups. The sample size is similar to published CMR studies that have investigated myocardial edema, myocardial hemorrhage and MVO in other clinical settings. TA use was left to operator discretion, which can lead to a selection bias that may impact outcomes. However, the lack of a trend towards a higher thrombus burden in the TA group suggests that most of the TA use was based on a routine use policy. Lastly, we used a surface coil which can decrease the sensitivity for detecting inferolateral edema and infarction with an opposite effect in the anteroseptal region, due to an inhomogeneous coil sensitivity profile.

## Conclusions

TA as an adjunctive therapy to primary PCI is associated with a reduction in the degree of myocardial edema, myocardial hemorrhage, infarct size, left ventricular remodeling, and MVO after STEMI. The positive impact of TA on myocardial edema, myocardial hemorrhage and left ventricular remodeling using CMR is a novel finding and therefore, we believe our data contribute important findings to the current literature on this topic. However, given the small sample size and non-randomized design the results of our study are hypothesis generating only and one cannot draw a causal relationship between favorable outcomes and use of TA in primary PCI. The results of large randomized trials to assess the utility of TA on the amount of salvaged myocardium using CMR are pending [53,54].

## Abbreviations

CMR: Cardiovascular magnetic resonance; LVEDVI: Left ventricular end diastolic volume index; LVESVI: Left ventricular end systolic volume index; LVSVI: Left ventricular stroke volume index; MVO: Microvascular obstruction; NTA: No thrombus aspiration; PCI: Percutaneous coronary intervention; STEMI: ST-segment elevation myocardial infarction; TA: Thrombus aspiration; TIMI: Thrombolysis in myocardial infarction

## Competing interests

Dr. Wright receives research funding from GE Healthcare.

## Authors' contributions

MIZ: Study design, patient identification, analysis and interpretation of data, statistics, drafting of the manuscript. NRG: Study design, analysis and interpretation of data, statistics, drafting of the manuscript. KAC: Study design, analysis and interpretation of data, statistics, revising the manuscript critically for important intellectual content. SBJ: Patient identification, analysis and interpretation of data, statistics, revising the manuscript critically for important intellectual content. BHS: Analysis and interpretation of data, revising the manuscript critically for important intellectual content. EAC: Analysis and interpretation of data, revising the manuscript critically for important intellectual content. GAW: Study design, analysis and interpretation of data, statistics, revising the manuscript critically for important intellectual content. AJD: Study design, analysis and interpretation of data, statistics, revising the manuscript critically for important intellectual content. All authors have read and approved the final manuscript.
